# Industry Perspectives on Market Access of Innovative Drugs: The Relevance for Oncology Drugs

**DOI:** 10.3389/fphar.2016.00144

**Published:** 2016-06-01

**Authors:** Kim Pauwels, Isabelle Huys, Minne Casteels, Steven Simoens

**Affiliations:** Department Pharmaceutical and Pharmacological Sciences, Clinical Pharmacology and Pharmacotherapy, Katholieke Universiteit LeuvenLeuven, Belgium

**Keywords:** drug development, market access, pricing and reimbursement, health technology assessment, oncology, health economics, qualitative research, industry

## Abstract

**Key Points**

- Representatives of the pharmaceutical industry call for a broader recognition of value within the assessment and appraisal of innovative drugs
- Focus on value within the assessment and appraisal of drugs is jeopardized by financial drives as the side of industry and at the side of the payers
- A well–considered value-framework, with attention for patient reported outcomes, societal preferences and dynamic approach on the drug life cycle, needs to be incorporated in assessment and appraisal at national and European level in order to coordinate the views of different stakeholders and allow efficient resource allocation

This study presents industry perspectives on the challenges related to market access of innovative drugs in general and oncology drugs in specific. Fifteen interviews were conducted with representatives of pharmaceutical companies and industry associations. Interviewees call for a broader recognition of value within the assessment and appraisal of drugs. According to interviewees, focus on value is jeopardized by the lack of a common value definition across Europe, poor availability and validity of value measures and cost-saving measures such as external reference price setting and cost-effectiveness analysis at the side of the payers. Centralized assessment of relative-effectiveness at European level would provide a common value estimate across member states, independent of financial drivers. Empirical evidence on PRO and societal preferences is however essential in the development of a value definition. Furthermore, value-based pricing would imply a dynamic approach where the price is differentiated across indications and across the lifecycle of the drug, especially in fields such as oncology. Financial drivers however also threat the application of value-based pricing at the side of the industry, making value-based profitability a more appropriate term.

## Introduction

In the aftermath of the economic crisis of 2010, public spending to health care is recovering slightly in line with the economic growth since 2013 (OECD, 2015). Spending to pharmaceuticals was cut by the introduction of cost containment measures in many countries and the end of the patents for some blockbuster drugs (OECD, 2015). In times of budgetary constraints, health authorities and payers still keep struggling with new innovations that offer in their view too limited benefits at an unjustified high price. This is even more important for disease areas as oncology, in which high unmet needs remain. With more than 3 million new cases and 1.7 million deaths each year, cancer remains the second most important cause of death and morbidity in Europe (WHO, 2014). The oncology market is typically expanding by high cost drugs that offer relatively limited benefits in terms of overall survival (OS) or progression free survival (PFS) (Garattini and Bertele, 2002). Ipilimumab is one of the numerous examples of drugs that has recently been highly criticized as an over expensive drug since it was launched in UK at a price of £75,000 for a four dose treatment course, yielding 3.7 months in median overall survival in the phase 3 trial (NICE, 2012). Pharmaceutical companies are put under pressure and blamed for setting unsustainably high prices that threaten the accessibility of health care for patients (Experts in Chronic Myeloid Leukemia, 2013). While an upward trend in both the launch price of oncology drugs as well as the price per unit of health gain indeed raises questions about the future sustainability and accessibility of public health care systems (Garattini and Bertele, 2002; Howard et al., 2015), a decline in economic return for pharmaceutical companies possibly threatens future innovation (Berndt et al., 2015). The current pipeline is however well-filled with oncology agents, promising evolutions toward individualized therapies, immuno-oncology, and combination treatment schemes and thus predicting more hurdles to overcome (IMS Institute for healthcare informatics, 2015).

Marketing authorization is currently centralized at European level for most drugs, including oncology drugs, and involves a first step in assessment of the drugs' safety, quality, and efficacy irrespective of financial consequences or relative benefit compared to alternatives. Drug appraisal, comprising the value of the drug in relation to its competitors, is exclusively performed at the national level of the member states in the context of price setting and reimbursement decisions. Previous research already showed a variety of instruments for price setting and reimbursement of oncology drugs across European member states, leading to heterogeneity across and within countries, and poor transparency toward all stakeholders involved (McCabe et al., 2009; Pauwels et al., 2014). Efficient allocation of the scarce resources and sustainable development and access to innovative drugs can only be obtained when stakeholders coordinate their individual views as much as possible. The aim of this study is to reveal the perspective of the pharmaceutical industry on development and market access of innovative drugs in general and oncology drugs in particular, and discuss their proposals for future improvement.

## Methods

Semi-structured interviews were conducted with representatives of the pharmaceutical industry between August and November 2015. Interviewees were invited through e-mail and telephone based on purposive sampling. Companies with oncology drugs in their current or past product portfolio were selected. Additionally, two industry associations were included. Interviewees affiliated to these companies or associations were selected based on their knowledge and experience related to market access strategies, including marketing authorization, price setting, and reimbursement of (oncology) drugs. After searching the literature for evidence about industry perspectives on market access of drugs in general, an interview guideline was designed by selecting eight relevant statements from the literature (Table 1). The statements were discussed with the interviewees during face-to-face interviews or by telephone, dependent on their availability and preferences. Notes were taken during the interview and in case of permission of the interviewee, the interview was audio-recorded and *ad verbatim* transcribed. The grounded theory approach was applied to analyze the interviews using the qualitative data analysis software package Nvivo^®^. All transcripts were anonymized. Interviews were repeated until data saturation. All interviewees received the results section for final approval, while the remaining manuscript was written independently.

**Table 1:** **Statements from the literature for semi-structured interview guideline**.

“Manufacturers sometimes set research priorities on the basis of the pursuit of a research hypothesis, as opposed to developing new technologies that meet unmet social need” (Drummond et al., 2013)
“Resources for development of new technologies can be used much more effectively if industry and HTA agencies were to collaborate at an early stage in the development process” (Jöhnsson, 2015)
“The main actors in the health care sector have different perspectives on the value added by health technologies” (Drummond et al., 2013)
“Manufacturers feel that the restrictions on the use and price of health technologies resulting from HTAs limits their sales potential and ultimately the profits from which future research has to be funded” (Drummond et al., 2013)
“It has been discussed if the QALY fully captures the benefits of new cancer drugs. The higher cost per QALY criteria for oncology drugs may reflect a view that there may be an additional benefit or value of treatment that should be considered” (Jönsson, 2013)
“The question how to define justifiable prices for oncology drugs needs to be addressed. Currently, prices are often set in relation to international standards and investments of manufacturers” (Fibig, 2013)
“Value-based pricing is likely to give manufacturers an incentive to more closely align their R&D with social objectives” (Fibig, 2013)
“If all elements of value are considered and taken into account in decision making, there is a risk that higher prices will be the result” (Fibig, 2013)

## Results

Fifteen interviews were conducted, involving 13 pharmaceutical companies and two pharmaceutical industry association. Two of the fifteen interviews were conducted by telephone instead of face-to-face. Fourteen of the fifteen interviews were audio recorded.

### Research and development

It was previously suggested in literature that companies sometimes focus on research hypothesis instead of unmet medical or social needs (Drummond et al., 2013). Interviewees show divergent opinions on the role of medical and social need in the orientation of research and development of innovative drugs. While some interviewees agree that social or medical need has no impact or only becomes important at a later stage of development, others indicate that social and medical need is increasingly driving drug development.

“Patients are put in the first place, second we have to create trust amongst our payers, amongst our patients and physicians, and then, in the end the business will follow and the money will come…we have a commercial organization and in the end it is about revenues, that is correct, but we put patients in the first place.” (I1)

For some interviewees it is not completely clear what the health authorities' needs and priorities are.

“We have no clear guidance on the unmet needs that needs to be worked upon. Where are the priorities? They have not been put forward. So how can we address those unmet needs if we don't know what they are for the authorities?” (I2)

According to other interviewees, needs are perceived in function of willingness to pay related to pricing and reimbursement.

“A drug that does not bring added value will have lot of difficulties to get reimbursed. Today, there are already a lot of incentives to orient investment and research in function of a need.” (I3)

“I don't think that we would develop a technology that meets a need if nobody is going to pay for that need. Because there is no market, no opportunity for companies” (I4)

Some interviewees do not believe that willingness to pay can steer research and development into a certain direction.

“It would be possible to indicate the areas where there is a need, where it would be interesting to do research…but I don't believe that you can guide research…you need companies that do their research, they can come with a proposal, and then the society has the right to say it is not relevant enough, we don't pay for it.” (I5)

### Marketing authorization

Currently, national legislation and practice in European member states concerning price setting and reimbursement of drugs leads to a heterogeneity across Europe. Interviewees are in favor of a centralized assessment of the relative benefit at European level within the process of marketing authorization. This would make the focus for companies more clear as it can provide a common basis for assessment and appraisal of drugs across European member states.

“The assessment of relative efficacy and effectiveness based on solid science presents a set of core similarities across countries. It is therefore reasonable that greater collaboration would enhance efficiency and predictability for all stakeholders involved.” (I6)

### Price setting

Interviewees however hold the opinion that as long as the economic system is different for each country, the appraisal of pharmaceuticals needs to remain a national responsibility, and price must depend on the national budget and priorities.

“I don't believe that price setting in Europe needs to be homogenous….you need to differentiate across countries.…But currently it is transforming toward a national competence that is more embedded in Europe, and this is a good thing.” (I5)

Interviewees emphasize that prices should however not just allow to cover costs made in the past but more importantly, needs to ensure investments in the future pipeline.

*“First of all, you want to make a difference, second, you want to make money. Not just because you love to earn money, but because you would like to keep things going. We want to develop, we want to innovate. Of course you need to please shareholders and draw dividends and make profits but most important, you want to keep going the R*&*D.” (I7)*

Companies further fear they cannot meet the expectation of the payers. According to the interviewees, payers only show willingness to pay for breakthrough innovation, while interviewees are convinced that a step by step approach will lead to tremendous benefits at the long-term.

“I mean if companies would be able to just produce what payers are expecting, and just breakthrough…I think they would do it. If they don't, it is just because it doesn't work that way.” (I4)

It is indicated by interviewees that the focus on medical needs provokes a shift from pharmaceuticals for primary care to specialized medicines where the volume of sales is lower and therefore price needs to be higher to recoup the costs.

“The more specific you go, the less patients there are and therefore, the cost should sometimes be higher to recoup the investments.” (I8)

A number of countries currently applies external reference pricing (ERP) in order to set prices based on the price in a basket of other countries. This can lead toward harmonization of price levels. Furthermore, a price decrease in one particular country can have a tremendous effect on price levels in the basket of reference countries, and interviewees confirm that this can motivate pharmaceutical companies to keep list prices high.

“Also you have countries who use ERP, who create a system where prices are kept as high as possible by companies. I think that is an aspect of the situation that explains why oncology drug prices are kept as high as possible, and cannot be defined in just one way, or justified in just one way.” (I9)

It was emphasized that the inherent value of the drug is neglected within the system of ERP.

“The problem that we see today is that a lot of countries have this health technology assessment (HTA), but at the same time there is this ERP. What is the aim of having these data or having this discussion around which premium is appropriate when in the end you say I am not willing to pay more than my neighbor?” (I4)

Also taking into account cost-structure within price setting will not provide the right incentives according to the industry.

“Think in terms of incentives. If they pay your R&D costs, they say it doesn't matter what you develop, they will pay it anyway. The only right incentive is to pay a fair price for products they need, and that is value-based pricing. The government needs to say develop value and we will pay for it.” (I7)

### Clinical value

Companies value a drug based on the clinical, economic, and societal value. Interviewees indicate that the full value of the product is currently not sufficiently captured during the assessment and appraisal of drugs. At the clinical level, interviewees doubt whether OS and PFS cover the long-term benefits of treatments based on new mechanisms of action such as present in immuno-oncology.

“The clinical benefit scale captures todays clinical value. We look at the curve of PFS or OS, and basically we know that until 5 years value can be captured in clinical trial setting, but what can't be captured is the area after the curve that looks at the longer quality of survival. And this is not captured everywhere, and I suppose it is normal because these things that are coming to the market are new. There is work to be done to develop value mechanisms beyond the clinical trial setting, tools that allow to capture broader value. And really today what is being used is the median OS, or PFS. We think that this should be broader to really capture what is the benefit.” (I10)

Further, it is questioned by interviewees whether OS, PFS, and quality of life cover most relevant outcomes according to patients and physicians.

“The focus is recently very much on OS and quality of life but…I think there are other things that can define the value of the drug. How many patients can you treat with your drug? Is it a drug with a different mode of action? What can this bring to the patient and the physician? Is it interesting to have one more drug, without necessarily prolonging survival? Is it good to have one extra therapeutic option?” (I9)

Interviewees call for standardized, validated and recognized measures for patient reported outcomes (PRO).

“How do you measure quality of life or PRO in a way that gives you data that are recognized as valid?…There is no guidance or commonly agreed framework on how we should do it.” (I10)

### Societal value

At the economic and societal level, interviewees feel that the broader economic and societal value of a product, resulting from savings at the long term, such as recessed hospitalizations and absence from work, is often ignored by decision makers.

“Very often many countries don't include societal perspectives. So what about the treatment costs after cure? Very often you have to model or calculate costs during the disease period, but if you cure, the patients survive, what about the additional costs or savings after surviving?” (I11)

### Cost-benefit analysis

Cost-effectiveness analysis is introduced in a large number of European countries in order to evaluate the cost-benefit ratio of a drug in terms of an incremental cost-effectiveness ratio (ICER), expressed as cost per quality adjusted life year (QALY). This was recognized by interviewees as an appropriate tool to compare the costs and benefits of different treatments with each other but several remarks on the system where provided. Several interviewees argued that considering an ICER within price setting and reimbursement evaluations does not provide appropriate incentives to the pharmaceutical industry. According to the interviewees, the ICER motivates to develop in an area where existing treatments are expensive, because you can add up the price of the new technology to the existing price, resulting in a lower ICER compared to the situation where there is no (expensive) alternative treatment. Due to methodological requirements, costs per QALY will always be too high in domains where effectiveness is low and patient populations are small.

“In the field of unmet needs, you will be punished by the QALY system because you have no cost-reference. By considering cost per QALY, they say do not develop in an area where prices are low, do not develop in an area where there are no big gains in effectiveness expected and do not in an area where patient populations are small.” (I7)

Furthermore, disease specific outcome that are of high relevance for the patient are often not captured in QALY measures as these are mainly based on health outcomes measured with EQ5D, a standardized, five dimension scale developed by Euroqol.

“EQ5D does not capture everything, like hair loss is not captured, so hair loss has no influence on the QALY. Which is crazy because I can imagine that hair loss would have an impact on quality of life.” (I3)

### Implications for oncology drugs

Interviewees urge on a broader consideration of value through the lifecycle of the product especially when drugs are initially only used in late stage diseases, such as is often the case for oncology drugs. This is due to ethical considerations, where one tries to address the highest need and minimize potential risks for patients. This makes it hard to prove value in the initial stage of drug assessment and appraisal. Benefits will anyhow be larger at a later stage of market access when the indication has broadened.

“Once an oncology medicine is more established, it can be used in an adjuvant curative setting, as in patients with better prognosis, before tumors have spread. Measured outcome, by definition for such patients are often better than patients whose cancer is more advanced. Often, prices that were too high at initial assessment, can turn out cost-effective by the time the medicine is in an adjuvant or broader use.” (I6)

Additionally, recognition of value can mean restriction of the drug toward the most valuable indication.

“Personally I believe you should use the drugs where they are most appropriate. You should use them where they deliver the highest value. Where they deliver the highest value you can justify better your price than when they are used in an inappropriate way.” (I12)

Alternatively, if payers recognize value and are willing to apply value-based pricing, they would be ready to apply indication specific pricing, as the value for oncology products can definitely differ between indications.

“Now we have average prices per milligram. And you can have a drug in breast cancer, colorectal cancer, and adjuvant breast cancer and today this drug has the same price per milligram in all these indications. If we are in a society that is willing to pay for value, then the pricing model should allow for different prices per indication, as the value of the drug might be very different. This could make the discussion more acceptable for all stakeholders.” (I3)

### Belgium

Several issues related to the Belgian system for price setting and reimbursement of drugs were mentioned based on the experience of the interviewees with relation to price setting and reimbursement files concluded in Belgium in the past. In the Belgian law, therapeutic value is defined in function of efficacy, effectiveness, safety, applicability, and convenience for use. Therapeutic value is considered following the application for reimbursement, together with the price of the drug, the therapeutic and social need, and budget impact and cost-benefit ratio^1^. Interviewees are favorable to the law, but are critical of the application in practice. They have the impression that at the Belgian level, budgetary control currently overrules value assessment.

“I think the law goes very broad and specifies the five criteria that they use to define the added value of a drug. But in the end you always see that it boils down to the cost of your drug compared to the cost of the other ones. Adding quality of life, adding efficacy, adding a better safety for your drugs, and especially quality of life and safety aspects…usually they are not valued that high by the authorities.” (I1)

Cost-effectiveness analysis is mandatory for drug that claim added therapeutic value. Some interviewees perceive it only as an entry ticket to the procedure which is not further taken into account in the evaluation for reimbursement, since budget impact takes the lead. Other interviewees mention that even when your budget impact is relatively low, the ICER will be put forward as an issue.

“If you are not cost-effective you don't get reimbursement, but if you are cost-effective, the budget can be too high. Cost per QALY must be zero in Belgium before you can get reimbursement.” (I1)

Interviewees call for a better recognition of the patient perspective. In the current system, health insurance funds have the task to represent the patients, but at the same time they are responsible for the public health budget, which results in a dual role with inherent conflicts. This further strengthens budget impact as a deciding factor for reimbursement.

“Health insurance funds took the responsibility to defend the patient. They probably did that in good faith, but know they are payer as well as patient representative. That is a dual role, which is not easy.” (I5)

Companies show incomprehension toward the Belgian practice in which reimbursement for an additional indication is coupled with a price decrease. When an additional indication is approved for a drug that was already reimbursed, the company needs to compensate one third of the additional budget impact related to the new indication. This gives the impression toward the companies that the added value of this new indication is not taken into account.

“The rule in Belgium in very special. When you can show the budget impact with the new indication compared to budget impact that was existing without a new indication, one third of the increase in the budget should be taken by the company, meaning that you have to decrease the price of the product by X percent. …But maybe you can show really a therapeutic added value for the new indication, this will not be taken into account.” (I13)

Interviewees indicate that there is no fixed period of exclusivity anymore where companies can recoup their costs and this introduces new financial uncertainties to the companies. Companies will anticipate on price pressure and price erosions, leading to higher prices at initial price setting.

## Discussion

This study presents industry perspectives on the challenges related to market access of innovative drugs in general and oncology drugs in specific. Industry calls for a broader recognition of value within the assessment and appraisal of drugs. Pricing in function of value has the potential to stimulate valuable innovation since industry is eager to orient research and development of drugs in function of willingness to pay. The lack of a common value definition across European member states, poor availability, and validity of value measures, as well as financial responsibilities at the side of both industry and payer, however threat the application of value-based pricing.

The pharmaceutical landscape is currently changing. While in the early 90's massive entities were set up to profit from the economies of scale, companies currently focus on one particular area of strength (Gautam and Pan, 2015). This goes along with a move from drugs for primary care toward specialty drugs and biologicals for unmet needs (Gautam and Pan, 2015). While some companies hold on the principle of a supply driven market where payers need to deal with what industry brings on, a transition toward a demand driven market was observed in this study. Companies show willingness to orient their investments to areas of unmet needs, on the condition that the priorities are clearly defined and benefits in areas of unmet need will be rewarded. Companies have to tailor drug applications to the individual market requirements in European member states, evoking frustrations at the side of the company when objective measures, even those assessed at European level such as efficiency and side effects, are not treated the same way across member states. In the first step of drug assessment, regulators however only focus on the balance between benefits and risks to grant marketing authorization. Clinical superiority was only defined at European level in the context of EU orphan drug legislation in 2000. In 2008, the High Level Pharmaceutical Forum of the European Commission published core principles on clinical superiority including a definition on relative efficacy (RE) (Eichler et al., 2010). First pilot projects of The European Network for Health Technology Assessment (EUnetHTA), established in 2009 in order to work toward more effective use of resources in the HTA process, strived for a common HTA assessment report across European member states, presenting the core HTA information (EUnetHTA, 2015). An assessment of the external validity of national assessments of RE however resulted in a pessimistic view on potential harmonization (EUnetHTA, 2015). Although there appears to be a commonality between the use of RE at national level, the way that study design, outcomes and comparators were evaluated and accepted differs (EUnetHTA, 2015). Early dialogue can aid understanding of possible differences and will allow companies to consider the product development fit for regulatory purposes. Therefore, the Shaping Early Dialogue (SEED) consortium was set up in 2014 under supervision of Haute Autorité de Santé (HAS) in order to include 14 partners within 10 early dialogues between 2014 and 2015. Centralized assessment of RE is clearly not likely to be quickly achieved, but there is a growing belief from industry as well as regulators that EU assessment of RE can strength the EU market and is expected to trigger allocation of research and development resources away from me-too drugs toward drug development programs that aim superiority claims (Eichler et al., 2010; Bergmann et al., 2015). Centralized assessment of RE based on transparent grounds can enable focused clinical trial design with generation of relevant data (Bergmann et al., 2015). Costs will be lower and quality will be improved at the benefit of both industry and public health systems, on the condition that the assessment of RE is recognized and shared across MS. Standards for clinical trials will therefore raise when evidence needs to be reliable and applicable to all MS. While industry believes a centralized assessment of RE can harmonize and strengthen value consideration within the assessment by national health authorities, some authors doubt whether this would lead to enhanced access to cancer medicines as drug appraisal, related to health care costs, remains to be managed at national or local level (Bergmann et al., 2015).

At the side of the payers it is debated whether prices of drugs are still proportionally related to their benefits (Howard et al., 2015; IMS Institute for healthcare informatics, 2015). At the same time, pharmaceutical companies doubt whether current assessment and appraisal procedures capture the full value of drugs. Administrative pricing procedures such as ERP anyhow neglect the intrinsic value of the drug and although the number of countries that solely relies on this pricing method is limited, the influence on industry strategies and consequently medicine prices needs to be emphasized (Leopold et al., 2012a,b). ERP, together with the fact that parallel trade is allowed within the EU single market, can urge companies to artificially keep up the list price, which does not reflect confidential discounts and rebates. In countries such as Belgium, this can exemplify conflicting interest when the main focus of payers is on the budget, while the Belgian list price is used as a reference in a large number of other countries applying ERP. Industry perspectives indicate that the Belgian policy and practice fosters price setting away from the inherent drug value, as companies intend to anticipate on price erosions, budgetary pressures and an impact on the price in other countries due to ERP. A comparison between value-based pricing and EPR, conducted in the lap of the European Commission, was anyhow in favor of value-based pricing since EPR does not aim to reward (future) innovation (Kanavos et al., 2010). According to the EU commissions report, development of valuable drugs can only be motivated when assessments and appraisal consider a societal perspective in determination of costs and benefits associated with a new technology (Kanavos et al., 2010). Evidence about the societal worth of a broader benefit consideration is increasing. A study in 4118 citizens of Great Britain suggested a significant value for wider societal benefits (Linley and Hughes, 2012). Also a population study conducted in Belgium showed that the effect of innovations on current expenditures is valuable according to society (Cleemput et al., 2014). While siloed funding is applied among European countries, the Swedish Dental and Pharmaceutical Benefits Agency (TLV) is one of the sole exemptions where pricing and reimbursement decisions focus on value from the societal perspective instead of the health care perspective only (Kanavos et al., 2010). According to TLV guidelines, this means that all relevant costs and revenues irrespective of the payee should be considered, including cost of production loss and cost of increased survival as well as benefits to both caregivers and patients (TLV, 2003). Prices in Sweden are however among the highest in Europe and adoption of the societal perspective considerably increases the information requirements, so attention for accessibility in terms of time and price is an asset (Kanavos et al., 2010; Vogler et al., 2015). With regard to clinical benefits, research already suggested that societal valuation of quality of life is overruling the valuation of life expectancy when considering both the initial health state of the patients as well as treatment effects (Bryan et al., 2002; Schwappach, 2003). Several stakeholders currently call for a broader inclusion of PRO, referring to multi-dimensional measures of symptoms, quality of life, health status, treatment adherence, and treatment satisfaction. Demographic evolutions toward an aging population and more chronic diseases provide a context in which the relevance of outcomes related to the wellbeing of the patient, such as of quality of life in the broad sense is growing. By the end of 2014, a reflection paper discussing the use of PRO in oncology was added to the scientific guidelines for quality, safety, and efficacy requirements prepared by the European Medicines Agency's (EMA) Committee for Human Medical Products (CHMP) (EMA, 2014). In this reflection paper, the benefit of capturing personal and social context of the disease and treatment experience in addition to objective clinical measures such as OS and PFS was set against its challenges in setting the degree of clinically relevant differences, difficulties in sensitivity, and problems with feasibility in longitudinal studies (EMA, 2014). Results in lung cancer previously showed that similar outcomes where shown for PFS than for time to significant deterioration in tumor related responses as measured by PRO, questioning the added value of PRO (EMA, 2014). Validated tools to measure PRO, also including Health Related Quality of Life (HRQoL) measures are scarce. Weighting systems to link disease specific quality of life outcomes to utility scores are even lacking; challenging their use in cost-effectiveness analysis. Generic health classification systems such as EQ5D remain the single valid tool for QALY weighting, often based on population preferences since also at this level disease specific patient preferences are lacking.

Pricing in terms of clinical-effectiveness as well as cost-effectiveness, sets the costs and benefits of a new treatment against those of the existing alternatives and it was argued by Howard et al. that setting prices of new drugs based on prices of existing therapies is also against intrinsic value consideration, consistent with models for reference pricing (Howard et al., 2015). The benefit of the new drug can add up to the price of existing treatments, providing incentives for development in areas of highly priced existing therapies. Cost-effectiveness analysis in oncology is anyhow to the prejudice of industry since it is often applied immediately after launch, considering advanced, or palliative stages of the disease. The End-of-Life guidance was introduced in UK to adjust the cost-effectiveness threshold for late stage treatments. Although society attaches higher value to treatments for more severe disease, treatments for palliative diseases as such, are not valued higher than treatments for early stages of diseases (Linley and Hughes, 2012). In the Netherlands, the thresholds are applied in a flexible way, adjusting these to severity of disease (Pauwels et al., 2014). The evolutions in drugs development are however likely to further threaten the applicability of cost-effectiveness analysis as the relevance is somewhat questioned within small patient populations and combination therapies. Because the price per milligram is fixed across indications while value can differ, the actual cost-effectiveness also varies across indications. Oncology drugs are characterized by multiple indications with more than 50% of major cancer medicines marketed in 2014 were for multiple indications (IMS Institute for healthcare informatics, 2015; Mestre-Ferrandiz et al., 2015). Setting a price based on the indication shortly after launch can influence patient access during the life cycle of the drug. If the single price is based on higher value indications, the price might be higher than what is optimal for subsequent indications, leading to restricted access (Mestre-Ferrandiz et al., 2015). If the price is initially set for lower valued indications, which will often be the case for oncology drugs, this might discourage companies to further develop valuable indications (Mestre-Ferrandiz et al., 2015). As value changes over the life cycle of a drug, value-based pricing challenges a dynamic life cycle approach where price discrimination across indications is allowed. Managed entry agreements allow to set confidential contributions based on performance of the drug, even beyond clinical trial setting, promising a leading role for managed entry agreements for oncology drugs in the future. It is however doubted that industry is ready to set the price fully in relation to value as this will not lead to sufficient return in particular situations such as small indications, making value-based profitability a more appropriate concept than value-based pricing. Furthermore, besides a considerable responsibility in sustaining the provision of and access to innovative drugs, the financial responsibilities of health authorities toward the budget cannot be neglected. It remains tricky how to define value and link value with an acceptable price level for all stakeholders, involving an acceptable profitability toward the industry and reasonable budgetary impact toward the health authorities.

This study suffers of three main limitations. First, despite the fact that interviews were repeated until data saturation, a variety of opinions was observed across interviewees. The results of this study aim to reflect these differing opinions. Second, the experience of some of the interviewees was limited to the Belgian situation, while the results are applied to the European situation in general. Third, the experience of majority of the interviews refers to innovative drugs in general and not solely to oncology drugs, therefore results specific for oncology drugs are described separately.

## Conclusion

Value consideration is key, especially in expanding areas that face expensive therapies such as oncology. On the one hand value consideration can help to define priorities, pursue, and reward development of valuable drugs at the benefit of industry. On the other hand, a clear value definition can help to discriminate between therapies that offer marginal or substantial benefits at the side of the benefit of the payer. Focus on value is however jeopardized by financial drivers both at the side of the industry (return) and payers (budget). Centralized assessment of RE can provide a common value estimate across member states, independent of financial drivers. Given current demographic and scientific evolutions, re-consideration of the concept of value is however required and empirical evidence suggests that developments of validated measures for PRO and societal benefits are appropriate. At the level of the member states, payers needs to take account of contradictory incentives for industry that some cost-saving measures such as ERP and cost-effectiveness analysis can evoke.

## Author contributions

KP, IH, MC, and SS provided substantial contributions to the conception and design of the work. KP conducted the data acquisition analysis. KP, IH, MC, and SS were involved in the interpretation of data for the work. KP drafted the work and IH, MC, and SS revised it critically for important intellectual content. KP, IH, MC, and SS approved the final version to be published and are all accountable for all aspects of the work in ensuring that questions related to the accuracy or integrity of any part of the work are appropriately investigated and resolved.

### Conflict of interest statement

The authors declare that the research was conducted in the absence of any commercial or financial relationships that could be construed as a potential conflict of interest.

## Footnotes

^1^*Koninklijk Besluit tot Vaststelling van de Procedures, Termijnen en Voorwaarden Inzake de Tegemoetkoming van de Verplichte Verzekering voor Geneeskundige Verzorging en Uitkeringen in de Kosten van Farmaceutische Specialiteiten*. (2001). Available online at: http://www.ejustice.just.fgov.be/cgi_loi/change_lg.pl?language=nl&la=N&table_name=wet&cn=2001122138 (Accessed Aug 25, 2012).
